# How to Kill a Tapeworm in an Hour

**Published:** 1878-12

**Authors:** 


How to Kill a Tapeworm in an Hour.—Kousso and kamela are expensive drugs, nauseous to the taste, not always effectual, and requires several days to effect the death of the worm. Dr. Karl Bettelhiem, of Vienna, narrates in the *Deutches Archiv*, a heroic method and nearly sure cure in the short space of three-quarters of an hour or two hours. It is this: He inserts a tube in the œsophagus, to the stomach, and pours down from two hundred to four hundred grammes of very concentrated decoction of pomegranate root, having previously had his patient fast for twenty-four hours. The worm is stupefied and passed, head and all, to a certainty; the patient has no sickness of the stomach and no nauseous swallowing to do; and the drug is cheap.—*Medical Brief.*

